# Potential impact of omentin-1 genetic variants on perineural invasion in prostate cancer

**DOI:** 10.7150/jca.119089

**Published:** 2025-08-11

**Authors:** Wei-Chun Weng, Tien-Huang Lin, Xiu-Yuan He, Chia-Yen Lin, Hsi-Chin Wu, Yuan-Li Huang, Chao-Yang Lai, Chun-Hao Tsai, Yi-Chin Fong, Shun-Fa Yang, Chih-Hsin Tang

**Affiliations:** 1 Department of Post-Baccalaureate Medicine, College of Medicine, National Chung Hsing University, Taichung, Taiwan.; 2 Division of Urology, Department of Surgery, Tungs' Taichung Metroharbor Hospital, Taichung, Taiwan.; 3 Department of Nursing, Jenteh Junior College of Medicine, Nursing and Management, Miaoli, Taiwan.; 4 School of Post-Baccalaureate Chinese Medicine, Tzu Chi University, Hualien, Taiwan.; 5 Department of Urology, Buddhist Tzu Chi General Hospital Taichung Branch, Taichung, Taiwan.; 6 Department of Pharmacology, School of Medicine, China Medical University, Taichung, Taiwan.; 7 Division of Urology, Department of Surgery, Taichung Veterans General Hospital, Taichung, Taiwan.; 8 School of Medicine, Chung Shan Medical University, Taichung, Taiwan.; 9 School of Medicine, National Yang Ming Chiao Tung University, Taipei, Taiwan.; 10 School of Medicine, China Medical University, Taichung, Taiwan.; 11 Department of Urology, China Medical University Hospital, Taichung, Taiwan.; 12 Department of Urology, China Medical University Beigang Hospital, Beigang, Yunlin, Taiwan.; 13 Department of Medical Laboratory Science and Biotechnology, Asia University, Taichung, Taiwan.; 14 Department of Sports Medicine, College of Health Care, China Medical University, Taichung, Taiwan.; 15 Department of Orthopedic Surgery, China Medical University Hospital, Taichung, Taiwan.; 16 Institute of Medicine, Chung Shan Medical University, Taichung, Taiwan.; 17 Department of Medical Research, Chung Shan Medical University Hospital, Taichung, Taiwan.; 18 Chinese Medicine Research Center, China Medical University, Taichung, Taiwan.

**Keywords:** omentin-1, prostate cancer, genetic polymorphisms

## Abstract

One of the most prevalent cancers and a major global cause of mortality in men is prostate cancer (PCa). Omentin-1, an adipokine, has been shown to play a protective role by reducing proinflammatory cytokine secretion. The relationships among carcinogenic lifestyle factors, biochemical recurrence (BCR), *OMNT1* polymorphisms, and PCa remain unclear. We investigated the impact of clinicopathological features and four *OMNT1* gene variants on PCa risk in 701 Taiwanese male patients with and without BCR. Compared with the TT genotype, the TA+AA genotypes of SNP rs2274907 were associated with a lower risk of perineural invasion. Similarly, the AG+GG genotypes of rs4656959 were associated with a lower risk of perineural invasion compared to the AA genotype. Importantly, PCa patients without BCR exhibited the same effects. Interestingly, the wild-type TT homozygous genotype was associated with significantly lower *OMNT1* expression levels compared to the AA genotype of the rs2274907 variant. Additionally, *OMNT1* mRNA levels were lower in PCa tissues compared to normal tissues, indicating that omentin-1 acts as a protective factor in PCa.

## Introduction

One of the most prevalent cancers and a major global cause of mortality for men is prostate cancer (PCa). According to estimates, over 300,000 men in the US will receive a PCa diagnosis in 2025, and over 35,000 will pass away from the disease 1. Because it is asymptomatic in the beginning, PCa can be challenging to detect, underscoring the importance of monitoring for early signs. PCa is a very diverse disease that can range from slowly developing to extremely aggressive and deadly types 2. Metastatic disease is the primary cause of PCa-associated mortality. PCa typically shows a propensity to invade and expand along prostatic nerves, a condition recognized as perineural invasion (PNI), whereas tumor spread typically happens through blood arteries and lymphatic channels. From the prostate to the pelvic plexus, this invasion occurs 3. One unique microenvironment that has been found to promote PCa growth and dissemination is the perineural space 4. Additionally, research has connected PNI to a higher risk of biochemical recurrence (BCR) and higher surgical Gleason scores 5-7.

Adipokines are bioactive substances produced by adipose tissue that manage a variety of physiological functions, such as energy balance, insulin sensitivity, inflammation, and immune reactions. Key adipokines like adiponectin, leptin, apelin, and omentin-1 influence metabolic and inflammatory pathways through local or systemic actions via autocrine, paracrine, or endocrine mechanisms 8. In the context of cancer, adipokines exert complex influences by affecting the tumor microenvironment, cell growth, programmed cell death, formation of blood vessels, and invasion 9. Omentin-1 is a recently documented adipokine with 313 amino acids that is mostly produced in the small intestine and human omental and subcutaneous adipose tissue 10. Omentin-1 has anti-inflammatory and anti-insulin resistance properties 11. It has been demonstrated to provide a protective role in reducing proinflammatory cytokines secretion 12. Omentin-1 has been positively correlated with higher levels of anti-inflammatory cytokines, according to earlier experimental research 13, 14. In cancer, serum omentin-1 concentrations are inversely linked with obesity, suggesting that omentin-1 may serve as a marker of tumor progression 15. Furthermore, omentin-1 may function as a tumor-suppressor factor because renal cell carcinoma patients have been found to have reduced serum levels of omentin-1 16.

A difference in a single nucleotide that takes place at a particular location in the genome is recognized as a single nucleotide polymorphism (SNP) 17. SNP distribution frequency comparisons between patient populations are widely performed to forecast the risk and prognosis of diseases, such as cancer 18, 19. There is no information on the relationships between carcinogenic lifestyle variables and omentin-1 (*OMNT1*) gene polymorphisms and PCa. Therefore, this study examined how a cohort of Taiwanese men carcinogenic lifestyle variables and *OMNT1* gene polymorphisms affected their likelihood of developing PCa. SNPs in the *OMNT1* gene were examined in this study in relation to the risk of BCR and clinicopathological advancement in Taiwanese males with PCa who had undergone radical prostatectomy.

## Materials and Methods

### Study participants

Blood samples from 701 PCa patients who had robotic-assisted laparoscopic radical prostatectomy performed at Taichung Veterans General Hospital (TVGH; Taichung, Taiwan) between 2012 and 2018 were analyzed in this study. Before venous blood collection, all participants provided written informed consent, and the TVGH Institutional Review Board approved the study protocol (IRB no. CE19062A-2). Prostate-specific antigen (PSA) levels, pathologic Gleason grades, clinical and pathologic T (tumor) and N (node) staging, cancer invasion sites (seminal vesicle, perineural, and lymphovascular regions) 20, D'Amico classification, and the BCR status were among the medical information gathered at the time of diagnosis 21. Our patient cohort consisted of 479 individuals without BCR (PSA level ≥0.2 ng/mL, confirmed by a second test) and 222 individuals with BCR.

### Selection and genotyping of SNPs

Based on earlier studies in systemic lupus erythematosus, the *OMNT1* SNPs rs2274907, rs35779394, rs4656959, and rs79209815 were chosen 22. The minor allele frequencies for every SNP were more than 5%. QIAamp DNA Blood Kits (Qiagen, CA, USA) were performed to extract genomic DNA from 3 mL peripheral blood samples. The SNPs were subjected to allelic discrimination using previously outlined evaluation methods 23-25. RT-qPCR experiments and RNA isolation were conducted in accordance with our previously published protocols 24, 26, 27.

### Analysis of clinical dataset

To choose PCa patients from The Cancer Genome Atlas (TCGA), we performed an extra analysis. In order to identify PCa patients whose *OMNT1* gene expression was assessed in each tumor sample, *OMNT1* levels in PCa samples obtained from TCGA were examined 19. An extensive public resource for analyzing tissue-specific gene level and modulation is the GTEx portal (gtexportal.org/home/). It supplies quantitative trait loci (QTLs), histological images, and open-access gene expression data 28.

### Statistical analysis

The Fisher's exact test and the Mann-Whitney U test were used to examine the differences between the PCa and control groups; *p*-values of less than 0.05 were deemed statistically significant. Odds ratios (ORs) and their 95% CIs for correlations between genotype frequencies and PCa risk were computed performing logistic regression. The Statistical Analytic System (SAS) software, version 9.1 for Windows (SAS Institute Inc., CA, USA), was performed to analyze all of the collected data.

## Results

Clinical and demographic characteristics of both groups were assessed (Table 1). The groups did not differ in age in any noticeable way. Patients with BCR were significantly more likely to exhibit higher pathologic Gleason grades (3+4+5), advanced clinical T stages (T3/T4), advanced pathologic T stages (T3/T4), and pathologic N1 stage. Patients with BCR also presented with seminal vesicle invasion, perineural invasion, and lymphovascular invasion. Furthermore, a higher percentage of BCR patients were classified as high-risk according to the D'Amico risk classification.

Table 2 displays the genotyping results for the four *OMNT1* SNPs in the patients with and without BCR. The most prevalent alleles were homozygous T/T for rs2274907, rs35779394 and rs79209815, and homozygous A/A for rs4656959 (Table 2). None of the genotypes for the four *OMNT1* SNPs in the various groups showed significant relationships after controlling for pathologic Gleason grade group, clinical T stage, pathologic T stage, pathologic N stage, seminal vesicle invasion, perineural invasion, lymphovascular invasion and D'Amico classification (Table 2).

Moreover, we compared the data for these four *OMNT1* SNPs using 1000 Genomes and dbSNP study from the National Center for Biotechnology Information database. As shown in Table 3, the allele frequencies of *OMNT1* SNPs are consistent across these two databases and our study.

We then looked at how *OMNT1* gene polymorphisms affected the clinicopathologic traits of PCa patients. The TA+AA heterozygous genotypes (combined variant carriers) were linked to a markedly lower risk of perineural invasion to the TT genotype at rs2274907 (OR, 0.687; 95% CI, 0.485~0.972; *p*<0.05). In addition, the AG+GG heterozygote was linked to a lower risk of perineural invasion to the AA genotype at rs4656959 (OR 0.670; 95% CI, 0.476~0.944; *p*<0.05) (Table 4).

Furthermore, the combined variant carriers were linked to a lower risk of perineural invasion (OR 0.636; 95% CI, 0.431~0.938; *p*<0.05) in no BCR patients with the SNP rs2274907 than the TT wild-type (Table 5). Similarly, the AG or GG genotypes at rs4656959 were protective against the development of perineural invasion in no BCR patients compared to the AA genotype (OR 0.628; 95% CI, 0.427~0.922; *p*<0.05) (Table 6).

**Figure 1:**
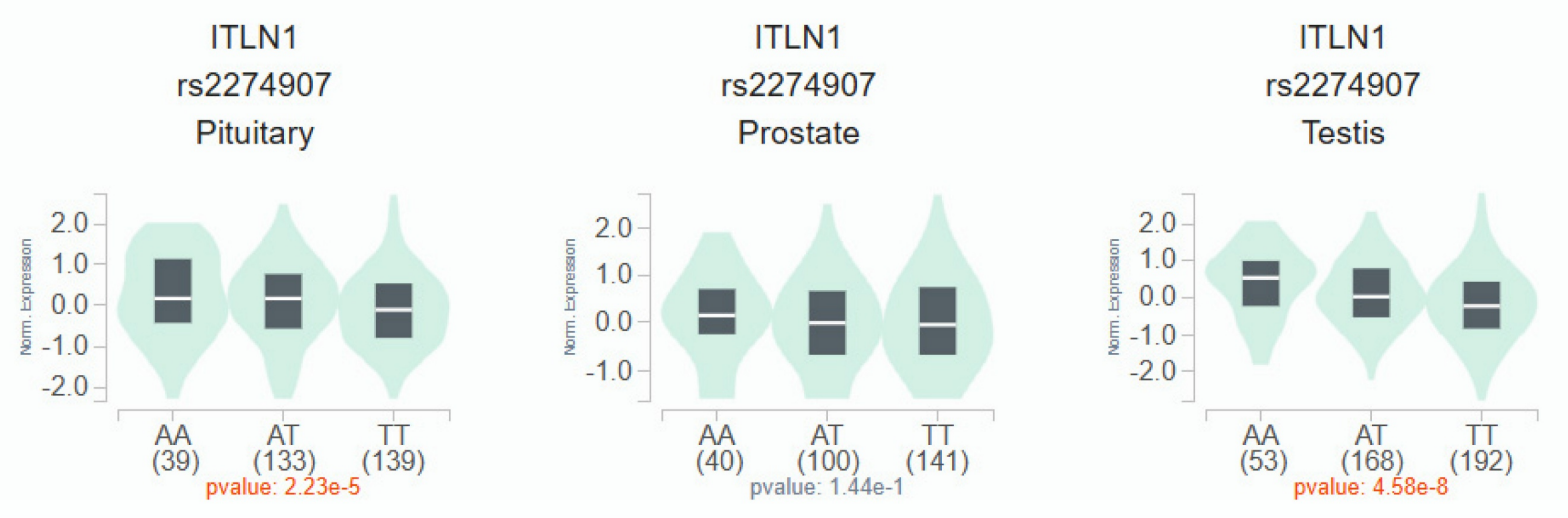
The *OMNT1* presents a significant eQTL association with rs2274907 genotypes in pituitary, prostate and testis from GTEx database.

**Figure 2:**
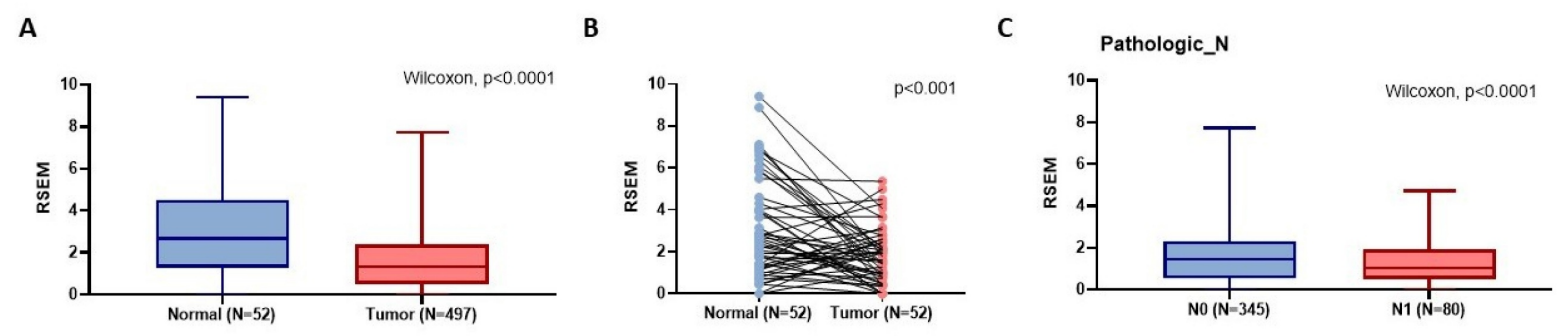
The *OMNT1* mRNA level of PCa patients from TCGA database. (A) The omentin-1 levels are lowered in PCa patient tissues compared to normal tissues from TCGA database. (B) The paired dot plot indicates that omentin-1 expression was lower in tumor tissues compared to paired normal tissues. (C) Omentin-1 expression levels in PCa patients from TCGA database were compared according to the pathologic N stage.

According to the GTEx data, patients with the wild-type TT homozygous genotype had considerably lower levels of *OMNT1* than those with the AA allele of variant rs2274907 in pituitary and testis tissues (*p*<0.05; Fig. 1) but not in prostate tissues (*p*=0.144; Fig. 1). Next, we examined *OMNT1* mRNA levels and their association with tumor stage in patients with PCa using the TCGA database. We found that tumor tissues exhibited lower *OMNT1* expression compared to normal tissues (Fig. 2A&B). Additionally, individuals with advanced pathologic N1 stage showed significantly lower *OMNT1* expression compared to those with N0 stage (Fig. 2C).

## Discussion

The effectiveness of tumor-related genetic aberration-based biomarkers in determining risk, facilitating early diagnosis, and forecasting treatment results has been validated by numerous cancer research investigations 29. About 1% of the general population has genetic polymorphisms, which are differences in genomic sequences between people. The most common alterations found in repetitive sequences are SNPs 30. The importance of SNPs and other genetic changes in predicting and defining pharmacotherapeutic effects in PCa has recently been highlighted by an expanding body of research 31, 32. We examined polymorphisms in the *OMNT1* gene, observing distinct distributions in PCa patients with and without BCR. Our analysis revealed that patients carrying the TA+AA genotypes of rs2274907 and the AG+GG genotypes of rs4656959 presented a markedly lower risk of developing perineural invasion, with stronger associations found in those without BCR. These results highlight the potential protective role of specific *OMNT1* genetic variants against perineural invasion, notably in PCa patients without BCR. Furthermore, we found that *OMNT1* expression levels were negatively linked with tumor growth and pathologic N1 stage in PCa patients.

Adipokines, a distinct bioactive peptide secreted by adipose tissues, are involved in many bodily functions 33. In order to ascertain how adipose tissue contributes to the development of inflammation and carcinogens, numerous researchers have been studying this topic for the past 20 years 34. Omentin-1 has recently been shown to play a crucial function in cell differentiation and accelerating cancer cell death 35. Numerous related research discovered that the circulation concentrations of omentin-1 in patients with colorectal and renal cell carcinoma varied, indicating that omentin-1 may have a role in the progression of cancer 36. On the other hand, little research has been done on omentin-1 and PCa. In order to compare the allelic distributions of four *OMNT1* gene polymorphisms between PCa patients with and without BCR, we conducted this study. We discovered that carriers of at least one A allele (combined variant carriers) at the *OMNT1* SNP rs2274907 and at least one G allele (AG+GG genotypes) at the *OMNT1* SNP rs4656959 were protected against developing perineural invasion. Importantly, PCa patients without BCR exhibited the same effects. Interestingly, GTEx data revealed that the wild-type TT homozygous genotype was associated with significantly lower *OMNT1* expression levels compared to the AA genotype of the rs2274907 variant in pituitary and testis tissues. The TCGA database confirmed consistent findings, showing that tumor tissues exhibit lower omentin-1 expression compared to normal tissues, and tumors at the advanced pathologic N1 stage have lower omentin-1 expression levels. Thus, omentin-1 may act as a negative regulator of PCa.

"Tumor metastasis" is the term used to describe the process by which the original tumor spreads via the blood or lymphatic system to other tissues or organs 37. It is crucial to comprehend the intricate process of metastasis since blocking angiogenesis and lymphangiogenesis may effectively stop tumor growth and metastasis 38-40. In addition to lymphatic and vascular routes, the neural route is a critical pathway for tumor spread, as perineural invasion can enhance the likelihood of regional or distant cancer dissemination 41. The process of tumor invasion into the perineural sheath is known as perineural invasion, and it significantly affects the prognosis of a number of cancers, including PCa 42, 43. According to our findings, patients carrying the TA+AA genotypes of rs2274907 and the AG+GG genotypes of rs4656959 have a markedly lower risk of developing perineural invasion, with stronger associations observed in those without BCR. These results suggest that the rs2274907 and rs4656959 genetic variants may restrict omentin-1 expression, thereby inhibiting perineural invasion and metastasis in PCa patients. To reinforce biological plausibility, rs2274907 and rs4656959 were investigated. rs2274907, located in an intron of IL3RA, overlaps an H3K27ac-marked enhancer in GM12878 cells and disrupts a predicted STAT3 binding site, potentially altering IL3RA expression in immune cells 44. rs4656959, located in an intron of CR1, is near a splice donor site and may affect mRNA stability through alternative splicing, consistent with its eQTL effect in brain tissues 45.

This study has several limitations that warrant discussion. False-Discovery-Rate (FDR) adjustment was not applied for key SNPs, as they were selected based on prior biological evidence, reducing the need for multiple testing correction 22. Without FDR adjustment, the reported *p*-values may include false positives, though the biological relevance of these SNPs supports their consideration. Additionally, several factors may explain the null association with BCR, including insufficient follow-up duration, the absence of interaction terms with perineural invasion, or omentin-1's primary role in regulating local tumor invasion rather than systemic recurrence.

In conclusion, our investigation is the first to reveal associations between *OMNT1* gene variants and perineural invasion in PCa patients. Our findings suggest that the *OMNT1* rs2274907 and rs4656959 variants protect against perineural invasion, particularly in PCa patients without BCR. Additionally, *OMNT1* mRNA levels were lower in PCa tissues compared to normal tissues, suggesting that omentin-1 acts as a negative regulator of PCa.

## Figures and Tables

**Table 1:** The distributions of demographical characteristics in 701 patients with prostate cancer.

Variable	biochemical recurrence (BCR)			
	No (n=479)	Yes (n=222)		p value
Age at diagnosis (years				
≤ 65	205 (42.8 %)		91 (41.0 %)	p=0.652
> 65	274 (57.2 %)		131 (59.0 %)	
Pathologic Gleason grade group				
1+2	350 (73.1 %)		70 (31.5 %)	p<0.001*
3+4+5	129 (26.9 %)		152 (68.5 %)	
Clinical T stage				
1+2	437 (91.2 %)		167 (75.2 %)	p<0.001*
3+4	42 (8.8 %)		55 (24.8 %)	
Clinical N stage				
N0	472 (98.5 %)		215 (96.8 %)	p=0.136
N1	7 (1.5 %)		7 (3.2 %)	
Pathologic T stage				
2	318 (66.4 %)		52 (23.4 %)	p<0.001*
3+4	161 (33.6 %)		170 (76.6 %)	
Pathologic N stage				
N0	467 (97.5 %)		174 (78.4 %)	p<0.001*
N1	12 (2.5 %)		48 (21.6 %)	
Seminal vesicle invasion				
No	434 (90.6 %)		117 (52.7 %)	p<0.001*
Yes	45 (9.4 %)		105 (47.3 %)	
Perineural invasion				
No	168 (35.1 %)		18 (8.1%)	p<0.001*
Yes	311 (64.9 %)		204 (91.9 %)	
Lymphovascular invasion				
No	445 (92.9 %)		144 (64.9 %)	p<0.001*
Yes	34 (7.1 %)		78 (35.1 %)	
D'Amico classification				
Low risk/Intermediate risk	272 (56.8 %)		75 (33.8 %)	p<0.001*
High risk	207 (43.2 %)		147 (66.2 %)	
				

* p value < 0.05 as statistically significant.

**Table 2:** Distribution frequency of *OMNT1* genotypes in 701 patients with prostate cancer.

Variable	biochemical recurrence (BCR)			
	No (n=479)	Yes (n=222)	AOR (95% CI)	p value
rs2274907				
TT	199 (41.5%)	96 (43.2%)	1.000 (reference)	
TA	233 (48.6%)	103 (46.4%)	1.030 (0.686-1.544)	p=0.888
AA	47 (9.9%)	23 (10.4%)	1.410 (0.721-2.756)	p=0.315
TA+AA	280 (58.5%)	126 (56.8%)	1.042 (0.858-1.265)	p=0.677
rs35779394				
TT	363 (75.8%)	157 (70.7%)	1.000 (reference)	
TC	105 (21.9%)	61 (27.5%)	1.207 (0.778-1.873)	p=0.401
CC	11 (2.3%)	4 (1.8%)	1.629 (0.370-7.168)	p=0.519
TC+CC	116 (24.2%)	65 (29.3%)	1.108 (0.894-1.374)	p=0.347
rs4656959				
AA	212 (44.3%)	106 (47.7%)	1.000 (reference)	
AG	223 (46.6%)	92 (41.4%)	0.871 (0.580-1.307)	p=0.504
GG	44 (9.1%)	24 (10.9%)	1.540 (0.794-2.986)	p=0.202
AG+GG	267 (55.7%)	116 (52.3%)	0.983 (0.811-1.191)	p=0.858
rs79209815				
TT	412 (86.0%)	179 (80.6%)	1.000 (reference)	
TC	62 (12.9%)	41 (18.5%)	1.399 (0.831-2.353)	p=0.206
CC	5 (1.1%)	2 (0.9%)	1.360 (0.172-10.731)	p=0.771
TC+CC	67 (14.0%)	43 (19.4%)	1.182 (0.916-1.524)	p=0.198

The adjusted odds ratios (AORs) with their 95% confidence intervals (CIs) were estimated by multiple logistic regression models after controlling for pathologic Gleason grade group, clinical T stage, pathologic T stage, pathologic N stage, seminal vesicle invasion, perineural invasion, lymphovascular invasion and D'Amico classification.

**Table 3:** Allele frequency of *OMNT1* single nucleotide polymorphisms (SNPs) in public database.

Study	Population/Ethnicity	Sample Size	*OMNT1* SNPs	
			rs2274907	
			T	A
This study	Taiwanese	701	66.05%	33.95%
1000Genomes	South Asian	978	62.80%	37.20%
dbSNP (NCBI)	Asian	168	64.90%	35.10%
			rs35779394	
			T	C
This study	Taiwanese	701	86.02%	13.98%
1000Genomes	South Asian	978	86.50%	13.50%
dbSNP (NCBI)	Asian	610	89.80%	10.20%
			rs4656959	
			A	G
This study	Taiwanese	701	67.83%	32.17%
1000Genomes	South Asian	978	65.60%	34.40%
dbSNP (NCBI)	Asian	2736	68.35%	31.65%
			rs79209815	
			T	C
This study	Taiwanese	701	91.65%	8.35%
1000Genomes	South Asian	978	95.50%	4.50%
dbSNP (NCBI)	Asian	128	96.90%	3.10%

**Table 4:** Odds ratios (ORs) and 95% confidence intervals (CIs) of the clinical status and *OMNT1* rs2274907 and rs4656959 genotypic frequencies in 701 patients with prostate cancer.

Variable	rs2274907				rs4656959			
	TT (*N*=295)	TA+AA (*N*=406)	OR (95% CI)	p value	AA (N=318)	AG+GG (N=383)	OR (95% CI)	p value
Pathologic Gleason grade group								
1+2	179 (60.7%)	241 (59.4%)	1.000	0.725	193 (60.7%)	227 (59.3%)	1.000	0.702
3+4+5	116 (39.3%)	165 (40.6%)	1.056 (0.778~1.435)		125 (39.3%)	156 (40.7%)	1.061 (0.783~1.437)	
Clinical T stage								
1+2	247 (83.7%)	357 (87.9%)	1.000	0.112	267 (84.0%)	337 (88.0%)	1.000	0.124
3+4	48 (16.3%)	49 (12.1%)	0.706 (0.460~1.086)		51 (16.0%)	46 (12.0%)	0.715 (0.465~1.098)	
Pathologic T stage								
2	148 (50.2%)	222 (54.7%)	1.000	0.238	156 (49.1%)	214 (55.9%)	1.000	0.072
3+4	147 (49.8%)	184 (45.3%)	0.834 (0.618~1.127)		162 (50.9%)	169 (44.1%)	0.760 (0.564~1.025)	
Pathologic N stage								
N0	268 (90.8%)	373 (91.9%)	1.000	0.632	291 (91.5%)	350 (91.4%)	1.000	0.953
N1	27 (9.2%)	33 (8.1%)	0.878 (0.516~1.495)		27 (8.5%)	33 (8.6%)	1.016 (0.597~1.730)	
Seminal vesicle invasion								
No	223 (75.6%)	328 (80.8%)	1.000	0.098	240 (75.5%)	311 (81.2%)	1.000	0.066
Yes	72 (24.4%)	78 (19.2%)	0.737 (0.512~1.059)		78 (24.5%)	72 (18.8%)	0.712 (0.496~1.023)	
Perineural invasion								
No	66 (22.4%)	120 (29.6%)	1.000	0.033*	71 (22.3%)	115 (30.0%)	1.000	0.022*
Yes	229 (77.6%)	286 (70.4%)	0.687 (0.485~0.972)		247 (77.7%)	268 (70.0%)	0.670 (0.476~0.944)	
Lymphovascular invasion								
No	242 (82.0%)	347 (85.5%)	1.000	0.221	266 (83.6%)	323 (84.3%)	1.000	0.805
Yes	53 (18.0%)	59 (14.5%)	0.776 (0.517~1.165)		52 (16.4%)	60 (15.7%)	0.950 (0.634~1.425)	
D'Amico classification								
Low risk/Intermediate risk	150 (50.8%)	197 (48.5%)	1.000	0.543	160 (50.3%)	187 (48.8%)	1.000	0.695
High risk	145 (49.2%)	209 (51.5%)	1.097 (0.813~1.481)		158 (49.7%)	196 (51.2%)	1.061 (0.788~1.429)	

ORs with their 95% CIs were estimated by logistic regression models. * p < 0.05 as statistically significant.

**Table 5:** Odds ratios (ORs) and 95% confidence intervals (CIs) of the clinical status and *OMNT1* rs2274907 genotypic frequencies in 701 prostate cancer patients with biochemical recurrence.

Variable	No biochemical recurrence (N=479)				biochemical recurrence (N=222)			
	TT (*N*=199)	TA+AA (*N*=280)	OR (95% CI)	p value	TT (*N*=96)	TT (*N*=126)	OR (95% CI)	p value
Pathologic Gleason grade group								
1+2	149 (74.9%)	201 (71.8%)	1.000	0.453	30 (31.3%)	40 (31.7%)	1.000	0.937
3+4+5	50 (25.1%)	79 (28.2%)	1.171 (0.775~1.770)		66 (68.8%)	86 (68.3%)	0.977 (0.552~1.731)	
Clinical T stage								
1+2	177 (88.9%)	260 (92.9%)	1.000	0.136	70 (72.9%)	97 (77.0%)	1.000	0.487
3+4	22 (11.1%)	20 (7.1%)	0.619 (0.328~1.168)		26 (27.1%)	29 (23.0%)	0.805 (0.436~1.485)	
Pathologic T stage								
2	127 (63.8%)	191 (68.2%)	1.000	0.316	21 (21.9%)	31 (24.6%)	1.000	0.634
3+4	72 (36.2%)	89 (31.8%)	0.822 (0.560~1.206)		75 (78.1%)	95 (75.4%)	0.858 (0.456~1.613)	
Pathologic N stage								
N0	194 (97.5%)	273 (97.5%)	1.000	0.993	74 (77.1%)	100 (79.4%)	1.000	0.682
N1	5 (2.5%)	7 (2.5%)	0.995 (0.311~3.181)		22 (22.9%)	26 (20.6%)	0.875 (0.460~1.663)	
Seminal vesicle invasion								
No	178 (89.4%)	256 (91.4%)	1.000	0.464	45 (46.9%)	72 (57.1%)	1.000	0.129
Yes	21 (10.6%)	24 (8.6%)	0.795 (0.429~1.471)		51 (53.1%)	54 (42.9%)	0.662 (0.388~1.129)	
Perineural invasion								
No	58 (29.1%)	110 (39.3%)	1.000	0.022*	8 (8.3%)	10 (7.9%)	1.000	0.915
Yes	141 (70.9%)	170 (60.7%)	0.636 (0.431~0.938)		88 (91.7%)	116 (92.1%)	1.055 (0.400~2.782)	
Lymphovascular invasion								
No	183 (92.0%)	262 (93.6%)	1.000	0.498	59 (61.5%)	85 (67.5%)	1.000	0.353
Yes	16 (8.0%)	18 (6.4%)	0.786 (0.390~1.581)		37 (38.5%)	41 (32.5%)	0.769 (0.442~1.340)	
D'Amico classification								
Low risk/Intermediate risk	115 (57.8%)	157 (56.1%)	1.000	0.708	35 (36.5%)	40 (31.7%)	1.000	0.462
High risk	84 (42.2%)	123 (43.9%)	1.073 (0.743~1.548)		61 (63.5%)	86 (68.3%)	1.234 (0.705~2.159)	

ORs with their 95% CIs were estimated by logistic regression models. * *p* < 0.05 as statistically significant.

**Table 6:** Odds ratios (ORs) and 95% confidence intervals (CIs) of the clinical status and *OMNT1* rs4656959 genotypic frequencies in 701 prostate cancer patients with biochemical recurrence.

Variable	No biochemical recurrence (N=479)				biochemical recurrence (N=222)			
	AA (*N*=212)	AG+GG (*N*=267)	OR (95% CI)	p value	AA (*N*=106)	AG+GG (*N*=116)	OR (95% CI)	p value
Pathologic Gleason grade group								
1+2	157 (74.1%)	193 (72.3%)	1.000	0.664	36 (34.0%)	34 (29.3%)	1.000	0.456
3+4+5	55 (25.9%)	74 (27.7%)	1.094 (0.728~1.645)		70 (66.0%)	82 (70.7%)	1.240 (0.704~2.187)	
Clinical T stage								
1+2	189 (89.2%)	248 (92.9%)	1.000	0.151	78 (73.6%)	89 (76.7%)	1.000	0.588
3+4	23 (10.8%)	19 (7.1%)	0.630 (0.333~1.190)		28 (26.4%)	27 (23.3%)	0.845 (0.459~1.555)	
Pathologic T stage								
2	134 (63.2%)	184 (68.9%)	1.000	0.189	22 (20.8%)	30 (25.9%)	1.000	0.369
3+4	78 (36.8%)	83 (31.1%)	0.775 (0.529~1.134)		84 (79.2%)	86 (74.1%)	0.751 (0.401~1.405)	
Pathologic N stage								
N0	206 (97.2%)	261 (97.8%)	1.000	0.685	85 (80.2%)	89 (76.7%)	1.000	0.531
N1	6 (2.8%)	6 (2.2%)	0.789 (0.251~2.483)		21 (19.8%)	27 (23.3%)	1.128 (0.645~2.336)	
Seminal vesicle invasion								
No	188 (88.7%)	246 (92.1%)	1.000	0.198	52 (49.1%)	65 (56.0%)	1.000	0.298
Yes	24 (11.3%)	21 (7.9%)	0.669 (0.361~1.238)		54 (50.9%)	51 (44.0%)	0.756 (0.445~1.282)	
Perineural invasion								
No	62 (29.2%)	106 (39.7%)	1.000	0.017*	9 (8.5%)	9 (7.8%)	1.000	0.842
Yes	150 (70.8%)	161 (60.3%)	0.628 (0.427~0.922)		97 (91.5%)	107 (92.2%)	1.103 (0.421~2.892)	
Lymphovascular invasion								
No	196 (92.5%)	249 (93.3%)	1.000	0.733	70 (66.0%)	74 (63.8%)	1.000	0.726
Yes	16 (7.5%)	18 (6.7%)	0.886 (0.440~1.781)		36 (34.0%)	42 (36.2%)	1.104 (0.635~1.917)	
D'Amico classification								
Low risk/Intermediate risk	121 (57.1%)	151 (56.6%)	1.000	0.909	39 (36.8%)	36 (31.0%)	1.000	0.365
High risk	91 (42.9%)	116 (43.4%)	1.021 (0.710~1.470)		67 (63.2%)	80 (69.0%)	1.294 (0.741~2.258)	

ORs with their 95% CIs were estimated by logistic regression models. * *p* < 0.05 as statistically significant.

## References

[1] Corn BW, Feldman DB (2025). Cancer statistics, 2025: A hinge moment for optimism to morph into hope?. CA Cancer J Clin.

[2] Adamaki M, Zoumpourlis V (2021). Prostate Cancer Biomarkers: From diagnosis to prognosis and precision-guided therapeutics. Pharmacol Ther.

[3] Zareba P, Flavin R, Isikbay M, Rider JR, Gerke TA, Finn S (2017). Perineural Invasion and Risk of Lethal Prostate Cancer. Cancer epidemiology, biomarkers & prevention: a publication of the American Association for Cancer Research, cosponsored by the American Society of Preventive Oncology.

[4] Ayala GE, Dai H, Ittmann M, Li R, Powell M, Frolov A (2004). Growth and survival mechanisms associated with perineural invasion in prostate cancer. Cancer Res.

[5] Kraus RD, Barsky A, Ji L, Garcia Santos PM, Cheng N, Groshen S (2019). The Perineural Invasion Paradox: Is Perineural Invasion an Independent Prognostic Indicator of Biochemical Recurrence Risk in Patients With pT2N0R0 Prostate Cancer? A Multi-Institutional Study. Advances in radiation oncology.

[6] Ball MW, Partin AW, Epstein JI (2015). Extent of extraprostatic extension independently influences biochemical recurrence-free survival: evidence for further pT3 subclassification. Urology.

[7] Asadi-Samani M, Rafieian-Kopaei M, Lorigooini Z, Shirzad H (2018). A screening of growth inhibitory activity of Iranian medicinal plants on prostate cancer cell lines. BioMedicine.

[8] Ouchi N, Parker JL, Lugus JJ, Walsh K (2011). Adipokines in inflammation and metabolic disease. Nature reviews Immunology.

[9] Grigoraș A, Amalinei C (2025). The Role of Perirenal Adipose Tissue in Carcinogenesis-From Molecular Mechanism to Therapeutic Perspectives. Cancers.

[10] Schäffler A, Neumeier M, Herfarth H, Fürst A, Schölmerich J, Büchler C (2005). Genomic structure of human omentin, a new adipocytokine expressed in omental adipose tissue. Biochim Biophys Acta.

[11] Li Z, Liu B, Zhao D, Wang B, Liu Y, Zhang Y (2017). Omentin-1 prevents cartilage matrix destruction by regulating matrix metalloproteinases. Biomed Pharmacother.

[12] Rao SS, Hu Y, Xie PL, Cao J, Wang ZX, Liu JH (2018). Omentin-1 prevents inflammation-induced osteoporosis by downregulating the pro-inflammatory cytokines. Bone Res.

[13] Lin YY, Huang CC, Ko CY, Tsai CH, Chang JW, Achudhan D (2025). Omentin-1 modulates interleukin expression and macrophage polarization: Implications for rheumatoid arthritis therapy. Int Immunopharmacol.

[14] Ko CY, Lin YY, Achudhan D, Chang JW, Liu SC, Lai CY (2023). Omentin-1 ameliorates the progress of osteoarthritis by promoting IL-4-dependent anti-inflammatory responses and M2 macrophage polarization. International journal of biological sciences.

[15] Kim JW, Kim JH, Lee YJ (2024). The Role of Adipokines in Tumor Progression and Its Association with Obesity. Biomedicines.

[16] Chinapayan SM, Kuppusamy S, Yap NY, Perumal K, Gobe G, Rajandram R (2022). Potential Value of Visfatin, Omentin-1, Nesfatin-1 and Apelin in Renal Cell Carcinoma (RCC): A Systematic Review and Meta-Analysis. Diagnostics (Basel, Switzerland).

[17] Chanock S (2001). Candidate genes and single nucleotide polymorphisms (SNPs) in the study of human disease. Dis Markers.

[18] Lu HJ, Chuang CY, Su CW, Chen MK, Yang WE, Yeh CM (2022). Role of TNFSF15 variants in oral cancer development and clinicopathologic characteristics. J Cell Mol Med.

[19] Chen KJ, Hsieh MH, Lin YY, Chen MY, Lien MY, Yang SF (2022). Visfatin Polymorphisms, Lifestyle Risk Factors and Risk of Oral Squamous Cell Carcinoma in a Cohort of Taiwanese Males. International journal of medical sciences.

[20] Epstein JI, Egevad L, Amin MB, Delahunt B, Srigley JR, Humphrey PA (2016). The 2014 International Society of Urological Pathology (ISUP) Consensus Conference on Gleason Grading of Prostatic Carcinoma: Definition of Grading Patterns and Proposal for a New Grading System. The American journal of surgical pathology.

[21] D'Amico AV, Whittington R, Malkowicz SB, Schultz D, Blank K, Broderick GA (1998). Biochemical outcome after radical prostatectomy, external beam radiation therapy, or interstitial radiation therapy for clinically localized prostate cancer. Jama.

[22] Zhang TP, Li HM, Li R, Zhang Q, Fan YG, Li XM (2020). Association of omentin-1, adiponectin, and resistin genetic polymorphisms with systemic lupus erythematosus in a Chinese population. Int Immunopharmacol.

[23] Lee HP, Chen PC, Wang SW, Fong YC, Tsai CH, Tsai FJ (2019). Plumbagin suppresses endothelial progenitor cell-related angiogenesis in vitro and in vivo. Journal of Functional Foods.

[24] Lee HP, Wang SW, Wu YC, Tsai CH, Tsai FJ, Chung JG (2019). Glucocerebroside reduces endothelial progenitor cell-induced angiogenesis. Food and Agricultural Immunology.

[25] Wang B, Hsu CJ, Chou CH, Lee HL, Chiang WL, Su CM (2018). Variations in the AURKA Gene: Biomarkers for the Development and Progression of Hepatocellular Carcinoma. Int J Med Sci.

[26] Lee HP, Wu YC, Chen BC, Liu SC, Li TM, Huang WC (2020). Soya-cerebroside reduces interleukin production in human rheumatoid arthritis synovial fibroblasts by inhibiting the ERK, NF-kappa B and AP-1 signalling pathways. Food and Agricultural Immunology.

[27] Liu SC, Tsai CH, Wu TY, Tsai CH, Tsai FJ, Chung JG (2019). Soya-cerebroside reduces IL-1β-induced MMP-1 production in chondrocytes and inhibits cartilage degradation: implications for the treatment of osteoarthritis. Food and Agricultural Immunology.

[28] Carithers LJ, Moore HM (2015). The Genotype-Tissue Expression (GTEx) Project. Biopreserv Biobank.

[29] Vogelstein B, Papadopoulos N, Velculescu VE, Zhou S, Diaz LA Jr, Kinzler KW (2013). Cancer genome landscapes. Science.

[30] Allemailem KS, Almatroudi A, Alrumaihi F, Makki Almansour N, Aldakheel FM, Rather RA (2021). Single nucleotide polymorphisms (SNPs) in prostate cancer: its implications in diagnostics and therapeutics. American journal of translational research.

[31] Shiota M, Akamatsu S, Narita S, Terada N, Fujimoto N, Eto M (2021). Genetic Polymorphisms and Pharmacotherapy for Prostate Cancer. JMA journal.

[32] Hu SL, Liu SC, Lin CY, Fong YC, Wang SS, Chen LC (2024). Genetic associations of visfatin polymorphisms with clinicopathologic characteristics of prostate cancer in Taiwanese males. International journal of medical sciences.

[33] Taylor EB (2021). The complex role of adipokines in obesity, inflammation, and autoimmunity. Clinical science (London, England: 1979).

[34] Song YC, Lee SE, Jin Y, Park HW, Chun KH, Lee HW (2020). Classifying the Linkage between Adipose Tissue Inflammation and Tumor Growth through Cancer-Associated Adipocytes. Molecules and cells.

[35] Zhang YY, Zhou LM (2013). Omentin-1, a new adipokine, promotes apoptosis through regulating Sirt1-dependent p53 deacetylation in hepatocellular carcinoma cells. European journal of pharmacology.

[36] Shen XD, Zhang L, Che H, Zhang YY, Yang C, Zhou J (2016). Circulating levels of adipocytokine omentin-1 in patients with renal cell cancer. Cytokine.

[37] Lei PJ, Fraser C, Jones D, Ubellacker JM, Padera TP (2024). Lymphatic system regulation of anti-cancer immunity and metastasis. Front Immunol.

[38] Dieterich LC, Detmar M (2016). Tumor lymphangiogenesis and new drug development. Adv Drug Deliv Rev.

[39] Lin CY, Wang SW, Chen YL, Chou WY, Lin TY, Chen WC (2017). Brain-derived neurotrophic factor promotes VEGF-C-dependent lymphangiogenesis by suppressing miR-624-3p in human chondrosarcoma cells. Cell Death Dis.

[40] Lee HP, Wang SW, Wu YC, Lin LW, Tsai FJ, Yang JS (2020). Soya-cerebroside inhibits VEGF-facilitated angiogenesis in endothelial progenitor cells. Food Agr Immunol.

[41] Liebig C, Ayala G, Wilks JA, Berger DH, Albo D (2009). Perineural invasion in cancer: a review of the literature. Cancer.

[42] Amit M, Na'ara S, Gil Z (2016). Mechanisms of cancer dissemination along nerves. Nature reviews Cancer.

[43] Bakst RL, Wong RJ (2016). Mechanisms of Perineural Invasion. Journal of neurological surgery Part B, Skull base.

[44] Do C, Dumont ELP, Salas M, Castano A, Mujahed H, Maldonado L (2020). Allele-specific DNA methylation is increased in cancers and its dense mapping in normal plus neoplastic cells increases the yield of disease-associated regulatory SNPs. Genome biology.

[45] Deng N, Zhou H, Fan H, Yuan Y (2017). Single nucleotide polymorphisms and cancer susceptibility. Oncotarget.

